# Short and Long-Term Parental Posttraumatic Stress After a Child’s Accident: Prevalence and Associated Factors

**DOI:** 10.1007/s10578-019-00924-2

**Published:** 2019-09-07

**Authors:** Els P. M. van Meijel, Maj R. Gigengack, Eva Verlinden, Alida F. W. van der Steeg, J. Carel Goslings, Frank W. Bloemers, Jan S. K. Luitse, Frits Boer, Martha A. Grootenhuis, Ramón J. L. Lindauer

**Affiliations:** 1 Department of Child and Adolescent Psychiatry, Amsterdam UMC, University of Amsterdam, Meibergdreef 5, 1105 AZ Amsterdam, The Netherlands; 2 de Bascule, Academic Center for Child and Adolescent Psychiatry, Amsterdam, The Netherlands; 3 Pediatric Surgical Center of Amsterdam, Emma Children’s Hospital, Amsterdam UMC, University of Amsterdam & VU University, Amsterdam, The Netherlands; 4 Trauma Unit Department of Surgery, Amsterdam UMC, University of Amsterdam, Amsterdam, The Netherlands; 5 Department of Trauma Surgery, Amsterdam UMC, VU University, Amsterdam, The Netherlands; 6 Emergency Department, Amsterdam UMC, University of Amsterdam, Amsterdam, The Netherlands; 7 Pediatric Psychology Department of the Emma Children’s Hospital, Amsterdam UMC, University of Amsterdam, Amsterdam, The Netherlands; 8 Princess Maxima Center for Pediatric Oncology, Utrecht, The Netherlands

**Keywords:** Accident, Children and adolescents, Injury, Parent, Posttraumatic stress

## Abstract

Studies on the long-term prevalence of parental posttraumatic stress symptoms (PTSS) following child accidental injury are scarce, and findings on risk factors vary. In this follow-up study (T2, n = 69) we determined the prevalence of parental PTSS 2–4 years after accidental injury of their child, compared with 3 months after the accident (T1, n = 135). Additionally, we examined the association between parental and child factors and PTSS severity. Children were 8–18 years old at the time of the accident. Parent and child PTSS was assessed by self-report. Other data were retrieved from medical records and a telephone interview. Parental PTSS was 9.6% at T1 and 5.8% at T2. Acute parental stress as measured within 2 weeks of the child’s accident was significantly associated with parental PTSS severity (T1 and T2), as was the child’s hospitalization of more than 1 day at T1 and the child’s permanent physical impairment at T2. To prevent adverse long-term psychological consequences we recommend identifying and monitoring parents at risk and offering them timely treatment.

## Introduction

Accidental injury in children also affects the parents and puts them at risk for developing substantial posttraumatic stress symptoms (PTSS) [1, 2]. The prevalence of PTSS in parents 3–6 months after their child’s accidental trauma is 10–15%. In a preceding study, selfreported PTSS was measured in 135 parents, 3 months after their child’s accidental injury. Symptoms at a clinically significant level were reported by nearly 10% of the parents [3]. Kassam-Adams et al. [1] assessed self-reported PTSD in 251 parents of children with traffic-related injuries. They found partial or full posttraumatic stress disorder (PTSD) in 15% of the parents approximately 6 months post-injury. A systematic review on pediatric medical traumatic stress (PMTS) reported a prevalence of parental PMTS ranging from 0 to 18% at 10 months or more post-injury [4]. PMTS was defined as ‘a set of psychological and physiological responses of children and their families to pain, injury, serious illness, medical procedures, and invasive or frightening treatment experiences’, often including posttraumatic stress reactions [4]. While data are supportive for long-term PTSS and related impairment in parents [5], there is a lack of long-term follow-up studies. We only found one study with a 1 and 11 years follow-up period [6] in 48 mothers of children with burns. PTSS was assessed by self-report. At 1 year and 11 years after their child’s burn event, 17% of the mothers reported clinically significant symptoms.

In general, parents’ well-being has an effect on the child’s functioning [7]. PTSS in parents, short and long-term, affects children in various ways. It is longitudinally related to poorer recovery of PTSS in the child [8]. Parental PTSS increases the risk of child PTSD [9] and parents’ early symptoms are a risk factor for persistent posttraumatic stress in injured children [10]. A meta-analysis reported significant effect sizes for the relationship between parent and child PTSS, suggesting that parental PTSS, especially maternal, may be a risk factor for child PTSS [11]. Authors of the Integrative Trajectory Model of Pediatric Medical Traumatic Stress [4] also stressed the role of parents following their child’s injury. The Integrative Trajectory Model of Pediatric Medical Stress provides a conceptual framework for traumatic stress responses across pediatric injuries and illnesses [4, 5]. The model is based on six assumptions. One of these is specifically relevant for understanding the role of parents: ‘a social ecological or contextual approach is optimal for intervention’. Their findings with that model suggest that parental PTSS increases risk for and maintenance of child PTSS. Parental PTSS not only affects the daily functioning of the parents themselves, but can also impact parenting practices and readiness to meet the demands of medical care for children [4]. The results of a qualitative study in parents following injury [12] suggest that a responsive parenting style supports child recovery. Parents report that their own distress interferes with the use of this parenting style [4, 12].

Given the probable adverse consequences for the parents as well as the children, it is important to identify parents at risk for high levels of posttraumatic stress as soon as possible after their child’s accident. Screening instruments such as the Screening Tool for Early Predictors of PTSD (STEPP) are suitable for this purpose [3, 13]. However, if the setting does not allow for the use of a screening instrument or if no screening method is available, other methods to identify parents at risk can be advisable. Therefore, insight into factors possibly associated with parental PTSS is necessary. Risk factors for adult PTSS or PTSD after their own trauma are well studied, but less is known about factors associated with parental posttraumatic stress reactions following child accidental trauma or injury [14]. Furthermore, studies on risk factors for parental PTSD usually involve mixed populations of ill and injured children, and risk factors across these groups appear to vary [4]. Factors associated with parental posttraumatic stress can be parent-related or child-related. Prior trauma history is a consistent predictor of PTSD in adults following a subsequent trauma [15] and is a predictor of PTSD severity in parents of children with traffic-related injuries [1]. Acute stress responses in parents of children treated in the pediatric intensive care unit were found to be related to parental PTSD [16], and peritraumatic distress was found to be a predictor of PTSD in mothers of victims of motor vehicle accidents [17]. Witnessing the event was associated with parental PTSD [18], but parents can be at risk for PTSD even if they are not directly involved in their child’s accident [10]. The number of initial days in hospital significantly predicted PTSS (short and long-term) in parents of a mixed population of accidentally injured children and children with diabetes and cancer [8]. However, Bronner et al. [16] found no predictive value for the length of hospital stay in parents of children that received unexpected intensive care treatment. To date, severe pain in children and permanent physical impairment of injured children have not been studied in relation to parental PTSS. Obviously, parents also experience stress watching their child having severe pain. Furthermore, permanent physical impairment of children is likely to have impact on the parents, possibly comparable to the impact of extensive permanent scarring on parents of children with burns [6].

Regarding prevention of chronic posttraumatic stress, trauma-focused psychotherapy has been shown to be effective and is highly recommended by the National Institute for Clinical Excellence (NICE) [19]. However, de Vries et al. [18] stated that only 20% of the parents with PTSS seek help for themselves. Given the adverse effect of parental PTSS and the positive effect of trauma-focused psychotherapy, it would be useful to know more about the choices of parents regarding psychotherapy. This information could be of help in providing support, psycho-education or interventions to parents following their child’s accidental injury and could potentially clarify the relationship of psychotherapy with long-term posttraumatic stress.

The overarching aim of this study was to contribute to the knowledge of short and long-term parental posttraumatic stress following child accidental injury. In our study we therefore aimed to: (1) determine the long-term prevalence of PTSS in parents, 2–4 years after accidental injury of their child, compared with 3 months after the accident; (2) describe the association between parent prior trauma history, acute parental stress, witnessing the child’s accident, new traumatic events, child’s hospitalization, child’s severe pain and permanent physical impairment, and the severity of parental PTSS; (3) survey the choices of parents regarding trauma-focused psychotherapy. In line with previous research we hypothesized the following: (1) the prevalence of parental PTSS does not change over time unless parents completed trauma-focused psychotherapy; (2) there is a positive relationship between parent prior trauma history, acute parental stress, witnessing the child’s accident, new traumatic events, child’s hospitalization, child’s severe pain and permanent physical impairment and severity of parental PTSS and (3) a minority of the parents choose trauma-focused psychotherapy for themselves.

## Methods

### Procedure

From 2008 to 2010, we conducted a study in which we evaluated the Screening Tool for Early Predictors of PTSD (STEPP), a screening instrument to determine the risk of PTSD in children aged 8–18 who had been injured due to accidental trauma and in their parents [3]. This study was concluded with the assessment of posttraumatic stress in the children and in one of each child’s parents 3 months after the child’s accident (T1). The design of that study did not include a follow-up assessment. However, in 2012–2013, we had the opportunity to conduct a follow-up assessment in a limited period of time. Despite resulting variability due to the range of 2 to 4 years in follow-up, we decided to perform this follow-up study. We contacted the families (the children and one of their parents) who had participated in the first study and we assessed PTSS in children and parents at 2 to 4 years after the accident (T2). The families first received a letter in which the follow-up study was announced and its purpose was explained. Subsequently, we contacted the families by telephone and invited them to participate in a telephone interview and to complete a questionnaire sent by email. Consent was given either in writing (by email) or during the initial telephone conversation (in which case this part of the conversation was audiotaped). The results of the child follow-up assessments are reported elsewhere [20]. Both studies were approved by the Medical Ethical Committees of the Academic Medical Center and VU University medical center, Amsterdam, the Netherlands.

### Participants

To answer the research questions in the present study, we used the data from both the STEPP study and the follow-up study as mentioned above. We excluded cases for which only child data, but no parental data, were available. From the STEPP study, data of 135 parents and children were available: 103 mothers (76.3%), 32 fathers (23.7%), 58 girls (43%) and 77 boys (57%). Of the 135 families participating in the STEPP study, 69 families (51.1%) participated in the follow-up study. Of the initial group, 29 families could not be reached (2 telephone numbers were no longer in use and 27 did not answer our calls) and 37 declined to participate. Reasons for declining participation were serious medical and/or psychological problems of the child (2 families) and lack of time or no interest (35 families).

### Measures

#### Factors Associated with Parental PTSS

**Fig. 1:**
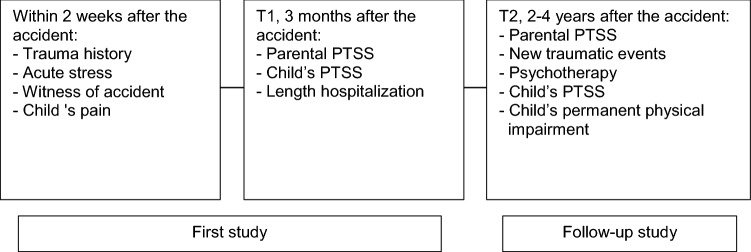
Summary of data collection

The multiple points of data collection are summarized in Fig. 1. Within 2 weeks after the accident the parents were asked the following closed questions (yes/no) on trauma history and acute stress: ‘Before the accident, did you ever experience anything frightening or horrible yourself?’ (Trauma history); ‘Have you felt very stressed or irritable since your child was injured or since your child has been in the hospital?’ (Acute stress); ‘If you now think about your injured child, do you perspire, shake or does your heart beat faster?’ (Acute stress). These questions were used in cooperation with the authors of the STEPP [13]. From the STEPP assessment we used the question: ‘Did you see the accident in which your child got hurt?’ Within 2 weeks after the accident we also asked children to rate the worst pain since the accident [21]. For this purpose, we used the Visual Analogue Scale (VAS), a small ruler with a 10-cm line, marked with *no pain* on the left and *the worst possible pain* on the right. The children used a sliding gauge to mark the location corresponding to the amount of pain they had experienced. The reverse side of the instrument shows the corresponding values from 0 to 100 mm. This instrument was used according to internal hospital guidelines [22]. Scores can be rounded to the nearest integer and categorized as *no or mild pain* (0–3), *moderate pain* (4–7) and *severe pain* (8–10). We used the category *children with severe pain* to examine the association with severity of posttraumatic stress of parents. Data on child hospitalization were derived from the medical records and checked with the parents at the 3-month assessment (T1). We divided the variable *length of hospitalization* into two categories: *hospitalization 1 day or less and hospitalization more than 1 day*.

The follow-up interviews (T2) started with the following open-ended questions, first regarding the child, and then regarding the parent him or herself: ‘How are things going?’ and ‘What has happened since we last met?’ With this initial part of the interview we aimed to become informed about the parents’ perception of the course of posttraumatic stress over time and about any other relevant health or mental health related information. Details on long-term consequences of the injury, specifically permanent physical impairment of children, were obtained from children and/or parents in this part of the follow-up assessment. In our study, permanent physical impairment was defined as loss or abnormality of parts of the body, resulting in restrictions or lack of ability to perform activities that were considered normal before the accident and are normal for children of that age. According to this definition, answers were coded dichotomously: the presence of permanent physical impairment ‘yes’ or ‘no’. Examples of permanent physical impairment are chronic or frequent pain, walking with a limp, partial deafness and chronic fatigue. Furthermore, a specific question was included regarding new traumatic events: ‘Since the accident, did other stressful things happen to you?’ DSM-IV-TR criteria for a traumatic event [23] were decisive for a positive or negative score on this item. Non-traumatic events were classified as life events. If the parent reported one or more new traumatic events, we asked how the parent felt about the consequences of the event and, if applicable, if help in any form was needed. Parents that reported PTSS at T1 or between T1 and T2, and parents that reported new traumatic or life events between T1 and T2, were asked if they had had any form of psychotherapy and if yes, we asked for more details about the therapy and the result of it.

#### Parental Posttraumatic Stress Symptoms (PTSS)

The parents completed a self-report instrument, the Dutch version of the Impact of Event Scale-Revised (IES-R) [24, 25]. The IES-R consists of 22 questions and contains the subscales re-experiencing, avoidance and hyperarousal [23]. An example of an item is: “I found myself acting or feeling like I was back at that time.” Scoring is on a 5-point scale. Items are rated according to the frequency of their occurrence during the past week (*Not at all* = 0, *A little bit* = 1, *Moderately* = 2, *Quite a bit* = 3, *Extremely* = 4; range 0–88). The focus is on the child’s accident. A total score of 23 or above indicates the likely presence of PTSD according to DSM-IV-TR criteria [23, 26]; in our study this was reported as PTSS, clinically significant posttraumatic stress. We used the total IES-R score to compare means between parents with and without PTSS and to test for associations with parental PTSS severity. A higher score indicates higher severity [26]. The Dutch IES-R showed adequate similarity with the total score of the Clinician-administered PTSD scale (CAPS; *r *= 0.75, *p *< .001) [26–28]. The internal consistency reliability (Cronbach’s alpha) of the current sample was 0.93.

#### Children’s Posttraumatic Stress

At T1, the children completed the Dutch version of the Child Revised Impact of Event Scale (CRIES) [29–31]. This self-report measure is based on the definition of PTSD according to DSM-IV-TR criteria and gives a good indication of the presence of PTSD [23, 31]. It consists of 13 questions in the subscales re-experiencing, avoidance and hyperarousal, with answers on a 4-point scale. An example of an item is: “Do you have waves of strong feelings about it?” We asked the children to focus on their accident when answering the questions. Items are rated according to the frequency of their occurrence during the past week (*Not at all *= 0, *Rarely *= 1, *Sometimes *= 3 and *Often *= 5). The Dutch CRIES is an effective and valid tool for screening of PTSD and shows moderate to good reliability: Cronbach’s alpha for the total score is 0.89 and for the subscales of re-experiencing, avoidance and hyperarousal 0.82, 0.77 and 0.74, respectively [31]. The total score can range from 0 to 65. The cut-off score for a positive test is 30. The outcome correlates highly with the PTSD diagnosis according to the Anxiety Disorders Interview Schedule for DSM-IV, Child and Parent Version (ADIS C/P) [31]. For the current sample Cronbach’s alpha was 0.87 [3]. In the current study we used a dichotomous variable: yes or no PTSS. PTSS is considered if symptoms are at a clinically significant level (a score of 30 or more) [31]. At T2, we used two self-report measures: the CRIES for children under 18 and the IES-R (see “Parental Posttraumatic Stress Symptoms” section above) for children 18 years and older.

### Data Analysis

We described parental and child characteristics using counts, percentages, means and standard deviations. Differences between follow-up participants and non-participants were analyzed with Mann–Whitney U tests for the continuous variable posttraumatic stress at the time of the first assessment, and a Fisher’s exact test for the categorical variable sex. In these tests, an alpha level of .05 was considered statistically significant.

We described the association between the level of parental PTSS and the level of child PTSS using Spearman’s correlation coefficient. We used univariable linear regression analysis to describe associations between the independent variables prior trauma history, acute parental stress, witnessing the accident, hospitalization of more than 1 day and severe pain, and the dependent variable parental PTSS severity as measured with the IES-R. We also added the independent variable permanent physical impairment of the child to the analyses of T2. We performed multivariable linear regression analysis using the independent variables with p < .10 in the univariable analysis. We then performed a backwards selection procedure until all independent variables had p < .05. Due to the skewed distribution of the PTSS data, we performed the linear regression analysis on log10 transformed data. To avoid taking the log10 of values of zero, we added one point to each parent’s score on the IES-R before performing the log10 transformation. To aid interpretation of the results, we back transformed the regression parameter estimates and corresponding upper and lower limits of confidence intervals. All analyses were performed using SPSS 24 (IBM Statistical Product and Service Solutions, Chicago, Ill).

## Results

### Participants

In the follow-up study we included 69 families, 58 mothers (84.1%), 11 fathers (15.9%), 28 girls (40.6%) and 41 boys (59.4%). The children had been exposed to various types of accidents: 43 (62.3%) had been involved in a traffic accident, 14 (20.3%) in a sports accident and 12 (17.4%) in other types of accidents, including falls.

There was no significant difference between follow-up participants and non-participants with regard to posttraumatic stress 3 months after the accident (U = 2082, Z = − .864, *p *= 0.39). There was a significant difference with regard to sex: fewer fathers than mothers completed follow-up (χ^2^ = 0.03, *p *= 0.04).

### Parental PTSS at T1 and T2

At T1, 122 parents reported no PTSS (90.4%; 92 mothers and 30 fathers) and 13 parents (9.6%; 11 mothers and 2 fathers) reported PTSS. Of these 13 parents, 9 were lost for follow-up: 5 of them declined participation due to lack of time or no interest, and 4 could not be reached. The mean IES-R score of parents with PTSS was 45.2 (SD 15.5, min–max 25–68); for parents without PTSS this was 5.6 (SD 5.4, min–max 0–21).

**Fig. 2:**
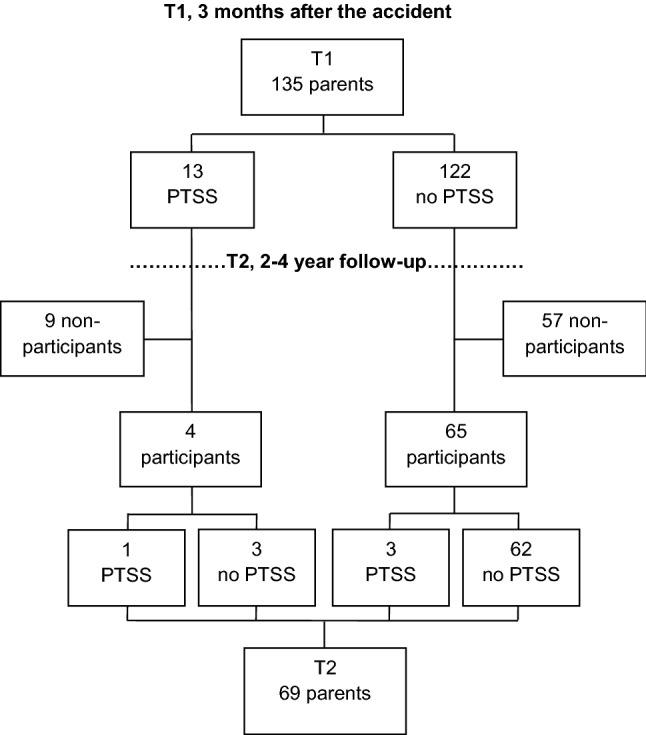
Parents with and without PTSS at T1 and T2

At T2, 65 parents reported no PTSS (94.2%; 54 mothers and 11 fathers) and 4 parents (5.8%; all mothers) reported PTSS. Of these four parents, one parent reported PTSS at T1 and three parents developed PTSS due to the accident between T1 and T2. The mean IES-R score of parents with PTSS was 34.3 (SD 10.6, min–max 24–49; for parents without PTSS this was 4.2 (SD 5.3, min–max 0–20). See also Fig. 2 for an overview.

We found a significant association between parental and child PTSS at T1 (Spearman’s ρ = 0.25, *p* < .001) but not at T2.

### Factors Associated with Parental PTSS

The univariable and multivariable associations between the parental and child factors of interest and the severity of parental PTSS are presented in Table 1 (T1) and Table 2 (T2). In both the univariable and the multivariable model, parental acute stress and hospitalization of more than 1 day of the child were significantly associated with severity of parental posttraumatic stress at T1. Parental acute stress and permanent physical impairment of the child were associated with parental PTSS severity at T2 in both the univariable model and the multivariable model.

**Table 1:** The univariable and multivariable associations between parent and child factors and the severity of parental PTSS (IES-R scores) at T1 (n = 135)

	Univariable model			Multivariable model		
	Beta^a^	95% confidence interval	*p* value	Beta^a^	95% confidence interval	*p* value
Parent characteristics						
Prior trauma history	1.35	0.91–2.00	0.13	–	–	–
Acute stress, irritable	2.07	1.44–2.98	0.000	1.60	1.11–2.29	0.01
Acute stress, physical	2.26	1.52–3.35	0.000	2.10	1.40–3.06	0.000
Witnessing accident	0.62	0.34–1.15	0.13	–	–	–
Child characteristics						
Hospital > 1 day	1.69	1.16–2.48	0.007	1.67	1.17–2.38	0.005
Severe pain	0.99	0.66–1.47	0.94	–	–	–

*T1* 3 months after the accident, *PTSS* clinically significant posttraumatic stress symptoms, *IES-R* Impact of Event Scale Revised

^a^Due to the skewed distribution of the parental PTSS data, we performed linear regression analysis on log10 transformed data. To aid interpretation of the results, we present back transformed regression parameter estimates and corresponding upper and lower limits of confidence intervals

**Table 2:** The univariable and multivariable associations between parent and child factors and the severity of parental PTSS (IES-R scores) at T2 (n = 69)

	Univariable model			Multivariable model		
	Beta^a^	95% confidence interval	*p* value	Beta^a^	95% confidence interval	*p* value
Parent characteristics						
Prior trauma history	0.93	0.54–1.61	0.80	-	–	–
Acute stress, irritable	1.78	1.05–3.01	0.03	1.68	1.00–2.81	0.048
Acute stress, physical^b^	1.71	0.95–3.07	0.07	–	–	–
Witnessing accident	0.59	0.28–1.27	0.17	–	–	–
Child characteristics						
Hospital > 1 day	1.49	0.86–2.57	0.15	–	–	–
Severe pain	0.77	0.45–1.34	0.36	–	–	–
Permanent physical impairment	2.51	1.44–4.40	0.002	2.16	1.23–3.81	0.008

*T2* 2–4 years after the accident, *PTSS* clinically significant posttraumatic stress symptoms, *IES-R* Impact of Event Scale Revised

^a^Due to the skewed distribution of the parental PTSS data, we performed linear regression analysis on log10 transformed data. To aid interpretation of the results, we present back transformed regression parameter estimates and corresponding upper and lower limits of confidence intervals

^b^Did not positively contribute to the multivariable level and was therefore left out

Two of the parents reported new traumatic events between T1 and T2: a life-threatening illness of a child and being involved in a car accident as a passenger. None of the two parents reported PTSS at T2. Due to the small number of parents that experienced a new traumatic event, this factor could not be included in the regression analysis. Of the remaining parents, 25 (37.3%) reported one or more life events but no traumatic events. Parents mentioned life events such as death or serious illness of a loved one, mostly one of their parents, concern about the mental or physical health of loved ones or of their own health, and becoming unemployed. Of the three parents who developed PTSS between T1 and T2, one parent reported a preceding stressful period (not specified) and one parent reported grief because of the death of her husband who died a few years before the child’s accident. Although parental PTSS was reported as a consequence of the accident, it could also have been influenced by grief or by a period of stress.

### Psychotherapy

Of the total group of 13 parents with PTSS at T1, 9 did not participate at T2. Of the remaining four parents with PTSS at T1, one parent still reported PTSS at T2 and three did not. The parent with PTSS at both T1 and T2 did not want any type of psychotherapy. She believed the symptoms would disappear over time. Of the three parents that no longer reported PTSS at T2, one still reported symptoms and distress but at a lower level. This parent started Eye Movement Desensitization and Reprocessing (EMDR) but did not finish it due to a mismatch with the therapist. The parent was willing to start EMDR again. The second parent successfully finished EMDR. The third parent did not want to be interviewed and only filled out the parent questionnaire, so it is unknown whether this parent received psychotherapy or not. The three parents that developed PTSS between T1 and T2 reported no need for psychotherapy. The first of these parents said that she didn’t need help and she would rather wait for recovery. If necessary, she would contact us at a later stage. The second parent said that she didn’t need trauma-focused therapy because she was already receiving general support from a social worker. The third parent stated that she didn’t need therapy because she only felt sad when talking about the accident.

## Discussion

The long-term prevalence of parental PTSS (5.8%) that we found in our study differs from the findings of previous studies on parental posttraumatic stress. Bronner et al. [16] studied parental PTSD in parents 9 months after unexpected pediatric intensive care unit (PICU) treatment of their child. The prevalence of clinical PTSD in their study was 10.5%. This percentage did not change over time, and posttraumatic stress responses at 3 months predicted subclinical and clinical PTSD at follow-up. Bakker et al. [6] studied maternal PTSS in children 1 and 11 years after a burn event of their child. Although mean total stress scores decreased significantly over time, 17% of the mothers reported clinically significant stress at both 1 year and 11 years after the burn event. There are several possible explanations for the discrepancy in findings with our study, such as the use of different questionnaires [16], different follow-up periods and different study populations. Furthermore, the majority of parents with PTSS at 3 months after the accident (9 out of 13) did not participate in the follow-up. If all 13 parents had participated in the follow-up assessment, it is likely that the prevalence at follow-up would have been higher and in agreement with assumptions based on previous studies [5, 6, 16].

In our study, parental and child posttraumatic stress were significantly associated 3 months after the accident, which is in line with the outcomes of other studies included in the meta-analysis of Morris et al. [11]. The association between child and parental PTSS, and the adverse effect of parental stress on the child’s PTSS and recovery, illustrate the important role of parental posttraumatic stress and the importance of adequate psychotherapy. In our sample, although it was very small, the majority of the parents reported no need for therapy. Our findings on the association between child and parent PTSS and the effects on children can be supportive in developing strategies to convince parents to accept adequate treatment.

In the univariable models, both acute stress items were significantly associated with the severity of parental PTSS at T1 and T2. In the multivariable models, this was the case at T1 but not at T2. At T2, one of the acute stress items did not contribute to the multivariable model. The differences between the multivariable models at T1 and T2 may result from the small sample size at T2 (n = 69). Our results show that acute parental stress is significantly associated with parental PTSS severity at 3 months and at 2–4 years post-injury. These results are in line with those of other studies [16, 17]. Furthermore, our results show that hospitalization longer than 1 day is associated with short and long-term parental PTSS severity. These findings are in line with those of Landolt et al. [8] but differ from those of Bronner et al. [16]. Our results also show that long-term permanent impairment of the child is associated with parental PTSS severity at follow-up. In future research, it might be useful to examine whether the length of hospitalization and later permanent impairment are related to the characteristics of the injury. If so, it might be possible to determine, at an early stage, what type of injury and/or what injury severity will probably lead to permanent impairment. Although injury severity itself is not a predictor for PTSS, research in children with burns indicates that there is an indirect relationship between burn extent and parental PTSS, through factors such as anxiety or guilt [6, 32].

This study had several limitations. First, almost half of the parents were lost to follow-up. Among those, 9 of 13 reported PTSS at 3 months. This precludes generalization and conclusions about the change of parental PTSS over time, as the estimated prevalence of PTSS at long-term follow-up may be biased. Second, the time between the first and follow-up assessment ranged from 2 to 4 years, resulting in variability in children’s development and transitions in life [20]. This could preclude generalization of the findings to other populations, specifically on the association between parental and child PTSS. Third, posttraumatic stress was assessed by questionnaire and not by clinical interview. Therefore, the prevalence of parental PTSS should be interpreted with caution. Fourth, acute stress (irritability), acute stress (physical), and trauma history were measured with only one question. Due to a lack of comprehensiveness, acute stress and trauma history may not have been adequately measured.

The present study adds to the knowledge of parental PTSS. The identification of factors associated with severity of later parental PTSS can support decisions about assessments and interventions in the various medical phases. In the peri-trauma and acute phase, special attention is required for the stress experienced by the parents, whether or not this is visible to the medical staff. Circumstances surrounding acute treatment of accidentally injured children are often unclear and therefore stressful for many parents. Medical staff should be trained to increase their awareness of acute parental stress, to prevent parental stress as much as possible, to ask about it systematically, to inform parents about it, and, if necessary, refer parents to a psychologist for intervention. To prevent interaction with the child’s response, parents can be helped in dealing with the circumstances and coping with their stress. Supporting parents to adequately address the child’s needs would facilitate child adjustment and recovery. Furthermore, to avoid persistent posttraumatic stress, we recommend timely screening for risk. Later on, systematic monitoring of parents of injured children is indicated, including screening for traumatic stress and treatment of significant traumatic stress. Overall, our results illustrate the importance of attention for parental posttraumatic stress to prevent adverse long-term psychological consequences for the parent and indirectly for the child. Further research is necessary to determine the prevalence of long-term PTSS in parents after accidental injury of their child and to confirm the role of factors associated with parental PTSS severity and their possible interaction.

## Summary

There are still gaps in the knowledge of parental PTSS following their child’s accident. In this follow-up study we therefore determined the prevalence of PTSS in parents 2–4 years after accidental injury of their child and described the association between parent and child factors and the severity of parental PTSS. Children were 8–18 years old at the time of the accident. Parent and child PTSS was assessed by self-report. Other data were retrieved from the medical records and from a telephone interview. Due to a high drop-out rate of parents with PTSS, the estimated long-term prevalence of 5.8% PTSS may be biased. Acute parental stress, the child’s hospitalization of more than 1 day, and the child’s permanent physical impairment were significantly associated with parental PTSS severity. To prevent adverse long-term psychological consequences for the parent—and indirectly for the child—we recommend identifying and monitoring parents at risk and offering them timely treatment. Special attention is required for parents with acute stress symptoms and parents with children at risk for permanent physical impairment.
